# Activated mammalian target of rapamycin is a potential therapeutic target in gastric cancer

**DOI:** 10.1186/1471-2407-10-536

**Published:** 2010-10-07

**Authors:** Da-zhi Xu, Qi-rong Geng, Ying Tian, Mu-yan Cai, Xin-juan Fang, You-qing Zhan, Zhi-wei Zhou, Wei Li, Ying-bo Chen, Xiao-wei Sun, Yuan-xiang Guan, Yuan-fang Li, Tong-yu Lin

**Affiliations:** 1 State Key Laboratory of Oncology in South China, Guangzhou 510060, China; 2 Department of Gastric & pancreatic Surgery, Cancer Center, Sun Yat-sen University, Guangzhou 510060, China; 3 Department of Medical Oncology, Cancer Center, Sun Yat-sen University, Guangzhou 510060, China; 4 Department of Pathology, Cancer Center, Sun Yat-sen University, Guangzhou 510060, China; 5 Department of Pathology, the Sixth Affiliated Hospital, Sun Yat-sen University, Guangzhou 510060, China

## Abstract

**Background:**

The mammalian target of rapamycin (mTOR) plays a key role in cellular growth and homeostasis. The purpose of our present study is to investigate the expression of activated mTOR (p-mTOR) in gastric cancer patients, their prognostic significance and the inhibition effect of RAD001 on tumor growth and to determine whether targeted inhibition of mTOR could be a potential therapeutic strategy for gastric cancer.

**Methods:**

The expression of p-mTOR was detected in specimens of 181 gastric cancers who underwent radical resection (R0) by immunohistochemistry. The correlation of p-mTOR expression to clinicopathologic features and survival of gastric cancer was studied. We also determined the inhibition effect of RAD001 on tumor growth using BGC823 and AGS human gastric cancer cell lines.

**Results:**

Immunostaining for p-mTOR was positive in 93 of 181 (51.4%) gastric cancers, closely correlated with lymph node status and pTNM stage. Patients with p-mTOR positive showed significantly shorter disease-free survival (DFS) and overall survival (OS) rates than those with p-mTOR-negative tumors in univariable analyses, and there was a trend toward a correlation between p-mTOR expression and survival in multivariable analyses. RAD001 markedly inhibited dose-dependently proliferation of human gastric carcinoma cells by down-regulating expression of p70s6k, p-p70s6k, C-myc, CyclinD1 and Bcl-2, up-regulating expression of P53.

**Conclusions:**

In gastric cancer, p-mTOR is a potential therapeutic target and RAD001 was a promising treatment agent with inducing cell cycle arrest and apoptosis by down-regulating expression of C-myc, CyclinD1 and Bcl-2, up-regulating expression of P53.

## Background

Gastric carcinoma is the fourth most common cancer and the second leading cause of cancer-related death worldwide, including 1 million new cases per year throughout the world [1]. About 60% of new cases of gastric cancer occur in eastern Asia [2], especially in China. Surgical resection is the most effective treatment for gastric cancer and the efficacy of chemotherapy remains limited [3]. Many studies have indicated that the depth of invasion and the number of metastatic lymph nodes are the most important powerful predictors of survival for gastric cancer patients [4,5]. For example, in previous studies [6,7], we have found positive lymph node ratio is an independent prognostic indicator after D2 resection and intraperitoneal chemotherapy may be beneficial to gastric cancer patients. However, a fundamental step toward improving clinical outcome lies in the increased understanding of the tumor biological behavior, which may help to identify the possible targets for individual therapy [8,9].

In recent years, molecularly based approaches for treatment in gastric cancer has been given great concern. Several articles have described some potential molecular targets for therapy in gastric cancer, such as epidermal growth factor receptor (EGFR) [10-13], vascular endothelial growth factor (VEGF) [14], recepteur d'origine nantais (RON) [15].

The mammalian target of rapamycin (mTOR) is a new potential molecular target for anticancer therapy. Its expression has been demonstrated in various cell lines and tumor specimens, such as liver neoplasms [16], breast cancer [17], biliary tract adenocarcinoma [18], and cervical cancer [19,20]. These studies showed that mTOR expression was associated with poor clinical prognosis. Lang et al [21]. have also observed mTOR activation in human gastric cancer in vitro and in vivo. They indicated mTOR is frequently activated in human gastric cancer. Recently, an orally bioavailable derivative of rapamycin, RAD001 (everolimus; Novartis Pharma AG), has been developed. A phase III trial revealed treatment with everolimus prolongs progression-free survival in patients with metastatic renal cell carcinoma that had progressed on other targeted therapies [22]. RAD001 has also been shown to inhibit the proliferation of tumor cell growth in some carcinomas [23,24]. But the role of RAD001 in gastric cancers cell is not clear.

The aims of this study were to further analyze the relationships the mTOR expression with the prognosis value of activated mTOR (p-mTOR), the effect of RAD001 on the growth and cell cycle of human gastric cancer cells in vitro and determine whether gastric cancer is a good candidate for target therapy with mTOR inhibitors.

## Methods

### Patients studied

This retrospective study consisted of 181 patients who underwent radical resection (R0) for histologically confirmed gastric carcinoma from Cancer Center of Sun Yat-sen University between January 1997 and December 2002. All patients with histologically confirmed adenocarcinoma of the stomach had undergone either proximal partial gastrectomy, distal partial gastrectomy or total gastrectomy. Ethical approval was obtained from Sun Yat-sen University Cancer Center research ethics committee.

Those with adequate histological material and complete clinical data were eligible for inclusion. The eligibility criteria also included histologically confirmed R0 resection, which was defined as no macroscopic and microscopic residual tumor and postoperative survival time ≥ 6 months. Patients with distant metastases and carcinoma of gastric stump after resection for benign disease were excluded from the study. The reason is that the survival of patients with distant metastases is often affected by many other factors, such as preoperative chemotherapy or obstruction.

All patients had follow-up after surgery at 6 to 12 month intervals; the final date of follow-up was December 2008. Median follow up period was 50 (mean: 50.66; range 10 to 128) months for all patients, and 74 months (mean: 72.80; range 27 to 128) for survival. Median duration of follow up was 40 (mean: 43.71; range 11 to 125) for patients who had tumors of p-mTOR positive expression, and 60.5 months (mean: 58.01; range 10 to 128) for negative expression. Survival was calculated from the date of diagnosis until the date of death or last follow-up.

### Specimen collection and Immunostaining for p-mTOR

All tumor specimens were obtained from surgically resected gastric cancers before adjuvant therapy. Formalin-fixed, paraffin-embedded tissue blocks were stored at room temperature identified by an identification number. Five-μm-thick tissue sections were cut from the paraffin blocks, deparaffinized in xylene, and rehydrated. Antigenic retrieval was processed with sodium citrate. The slides were then incubated in 0.3% H_2_O_2 _for 10 min, and blocked in 1% bovine serum albumin for 60 min followed by a rabbit monoclonal antibody specific for p-mTOR (Phospho-mTOR, Ser2448, 49F9; Cell Signaling Technology) at 4°C overnight, and then stained with 3,3-diaminobenzidine. After visualization of immunoreactivity, the sections were counterstained with hematoxylin and then mounted.

### Evaluation of Immunohistochemical Staining

The immunostained sections were evaluated and tissues from human prostate adenocarcinomas served as positive control. According to recently described criteria for rating mTOR expression, staining intensity was determined as 0 (absent), 1 (weak), and 2 (strong) and expression levels of the biomarkers were semiquantified using an immunohistochemistry score (range, 0-200) calculated by multiplying staining intensity with the percentage of positive tumor cells[18]. Patients with an immunohistochemistry score of ≤ 20 were considered as p-mTOR-negative and those with a score of > 20 as p-mTOR-positive. All slides were independently assessed by two independent pathologists without any knowledge of the patients' clinical information. When the opinions of the two evaluators were different, agreement was reached by careful discussion.

### Cell lines and culture conditions

Human gastric cancer cell lines BGC823 and AGS (ATCC Number: CRL-1739) which are poorly differentiated were gifts from Peking University School of Oncology (Beijing, P.R. China.) [25]. The cell lines were maintained in RPMI 1640 medium (Invitrogen) supplemented with 10% fetal bovine serum (Hyclone), penicillin (100 units/mL) and streptomycin (100 units/mL) at 37°C and 5% CO2 in a humidified incubator.

### Cell viability assay

To analyze the effect of RAD001 on human gastric cancer cells, cell viability was determined by MTT assay. RAD001 was provided by Novartis Pharma (Basel, Switzerland). For the viability assay, logarithmically growing cells were stimulated with various doses of RAD001 (0, 5, 10, 20 and 40 μM). At the end of culture period, 20 μL of tetrazolium dye 3-(4,5-dimethylthiazole-2-yl)-2,5- diphenyltetrazolium bromide (MTT) (Sigma, USA) solution at 0.2 mg/ml in PBS was added, the optical density at 490 nm (OD490) was determined by using an enzyme-linked immunosorbent assay (ELISA) reader. Mean values were calculated from triplicate cultures.

### Cell cycle analysis

Cells were collected and fixed in 70% ethanol overnight at 4°C. Cells were then washed and stained with propidium iodide (PI) (Sigma) 5 μg/mL. After 30 min at room temperature protected from light, the cells were analysed via flow cytometry using a Becton Dickinson FACScan.

### Annexin V assay

The samples were washed with phosphate-buffered saline (PBS) and using Annexin V-fluorescein isothiocyanate (FITC) and PI staining for determination of phosphatidylserine exposure on the outer plasma membrane. After incubation for 30 min at room temperature protected from light, the samples were quantified by flow cytometry, using a Becton Dickinson FACScan.

### Reverse transcription-PCR

Total RNA was extracted by using TRIzol method. RNA was reversely transcripted using a commercially available kit (Fermentas, USA). The mixture (25 μL total) for PCR consisted of 0.5 μl cDNA, 0.5 U Taq DNA polymerase, 2.5 μl of 10× PCR buffer, 2.5 mM dNTP mixture, and 50 pM sense and antisense primers each. CyclinD1 and C-myc were analyzed by following primers: CyclinD1 5'GAACAGAAGTGCGAGGAGGAG3' and reverse primer 5'AGGCGGTAGTAGGACAGGAAG3'; C-myc, 5' CGAGCTGCTGGGAGGAGACAT3' and 5' AGCCGCCCACTTTTGACA GG3' Actin: 5'GGCACCCAGCACAATGAA 3' and 5' TAGAAGCATTTGCGGTGG 3'.

### Cell lysate and Western blot analysis

Cells were lysed in lysis buffer and the concentration of protein was determined by the Bradford dye method (Bio-Rad Laboratories). Equal amounts of cell extract were subjected to SDS-PAGE and transferred to PVDF membrane (Bio-Rad). Western blot analyses were done with various specific primary antibodies. Antibodies recognizing phospho-p70S6K (Thr389), p70S6K, phospho-mTOR(Ser2448), Bcl-2 and p53 were from Cell Signaling.

### Statistical analyses

The primary end point of our study was overall survival (OS). It was defined as the period between the time of surgery and death. Disease-free survival (DFS) was the time between the time of surgery and relapse or tumor-related death. It was analyzed as a secondary end point. The association of p-mTOR expression with various clinicalopathologic features was analyzed using the chi-square test. Cumulative survival and disease-free survival were estimated by the Kaplan-Meier method. The log-rank test was used to evaluate the statistical significance of differences between survival curves. A Cox proportional hazard model (backward, stepwise) for multivariable analysis was applied for factors that achieved significance in univariable analysis. Statistical analysis was performed using SPSS software (version 13 for Windows; SPSS, Chicago, IL). Differences at P < .05 were considered to be statistically significant.

## Results

### Clinicopathologic features

A total of 181 patients were enrolled in this study. The median age was 59 years (range, 23-83 years). The clinicopathologic characteristics of the study population are summarized in Table 1. 129 patients were male and 52 female. 77 of 181 tumors (42.5) were from the upper stomach, 74 of 181 patients (40.9%) were from the lower part of the stomach, and the remaining tumors were from the middle and whole body of the stomach. 118 of 181(65.2%) patients' tumor size were > 4 cm, and 63 (34.8%) were ≤ 4 cm. In accordance with World Health Organization criteria, 4 of 181 tumors (2.2%) were well differentiated, 55 of 181 tumors (30.4%) were moderately differentiated, 116 were poorly differentiated, and 6 were undifferentiated. Post-operative surgical stage was classified according to the 2002 UICC/AJCC classification [26].

**Table 1:** Clinicopathologic correlation of p-mTOR expression in gastric cancer

Factors	All patients	No. of patients (%)		*P*
	*N *= 181(%)	mTOR negative	mTOR positive	
Age(y)				.256
< 60	95(52.5)	50(56.8)	45(48.4)	
≥60	86(47.5)	38(43.2)	48(51.6)	
Sex				.926
Male	129(71.3)	63(71.6)	66(71.0)	
Female	52(28.7)	25(28.4)	27(29.0)	
Site				.053
Upper	77(42.5)	33(37.5)	44(47.3)	
Middle	28(15.5)	10(11.4)	18(19.4)	
lower	74(40.9)	43(48.9)	31(33.3)	
Diffuse	2(1.1)	2(2.3)	0(0.0)	
Tumor size				.172
≤ 4 cm	63(34.8)	35(40.0)	28(30.1)	
> 4 cm	118(65.2)	53(60.0)	65(69.9)	
Borrmann type				.398
Early stage	7(3.9)	6(6.8)	1(1.1)	
I	4(2.2)	2(2.3)	2(2.1)	
II	65(35.9)	31(35.2)	34(36.6)	
III	101(55.8)	47(53.4)	54(58.1)	
IV	4(2.2)	2(2.3)	2(2.1)	
grading				.999
1	4(2.2)	2(2.3)	2(2.1)	
2	55(30.4)	27(30.7)	28(30.1)	
3	116(64.1)	56(63.6)	60(64.5)	
4	6(3.3)	3(3.4)	3(3.3)	
Pathologic T classification				.168
T1	17(9.4)	12(13.6)	5(5.4)	
T2	35(19.3)	18(20.5)	17(18.3)	
T3	119(65.7)	55(62.5)	64(68.8)	
T4	10(5.5)	3(3.4)	7(7.5)	
Pathologic N status				.008
N negative	44(24.3)	29(33.0)	15(16.1)	
N positive	137(75.7)	59(67.0)	78(83.9)	
Pathologic stage (pTNM)				.026
I	26(14.4)	17(19.3)	9(9.7)	
II	47(26.0)	28(31.8)	19(20.4)	
III	100(55.2)	41(46.5)	59(63.4)	
IV	8(4.4)	2(2.3)	6(6.5)	

129 of 181 patients (71.2%) had more advanced T stage disease (PT3+PT4), and 137 patients (75.7%) had lymph node metastasis. Totally patients of stage I-IV were 26, 47, 100 and 8 cases, respectively.

### p-mTOR expression in gastric cancer

**Figure 1:**
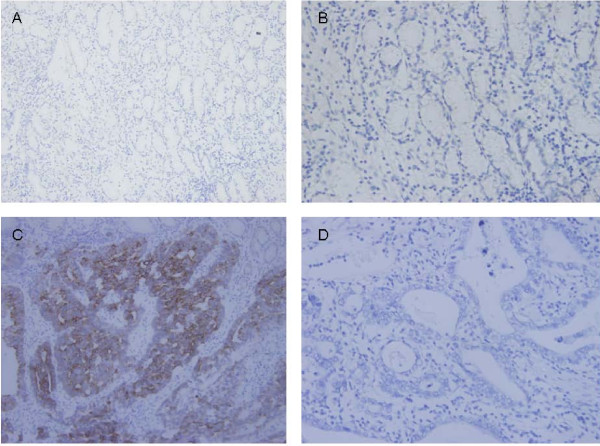
**Examples of p-mTOR immunostaining**. (A) The normal gastric mucosa is negative for p-mTOR (original magnification ×200). (B) The normal gastric mucosa is negative for p-mTOR (original magnification ×400) (C) Positive expression of p-mTOR in gastric cancer (original magnification ×200). (D) Negative expression of p-mTOR in gastric cancer (original magnification ×200).

Immunostaining of p-mTOR was cytoplasmatic and partly membranous. Figure 1 shows representative examples of p-mTOR immunostaining. The normal gastric mucosa is negative for p-mTOR. Of the 181 patients, 93 (51.4%) were p-mTOR-positive tumors.

### Survival analysis

**Figure 2:**
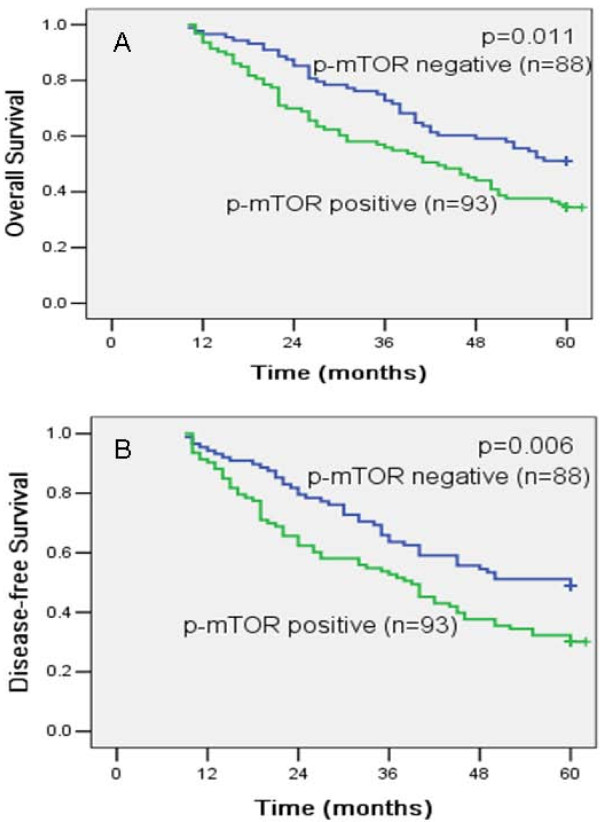
**Kaplan-Meier estimates of the probability of survival**. Patients with p-mTOR positive showed significantly shorter overall survival (A) and disease-free survival (B) rates than those with p-mTOR negative expression. (p = .011 and p = .006, respectively).

Patients with p-mTOR positive gastric cancer showed significantly shorter disease-free and overall survival rates than those with p-mTOR negative gastric cancer (DFS, 48.9% versus 30.1%; p = .006; OS, 51.1% versus 34.4%; p = .011; Table 2; Figure 2A, B). Univariable analysis revealed that survival time also decreased with higher pT classification (DFS, p = .001; OS, P = .005), lymph node metastasis (DFS, p < .0001; OS, P = .001) and advanced pTNM stage (DFS, p < .001; OS, P = .001). There was no association between DFS or OS and age, sex or tumor size.

**Table 2:** Univariable analysis of disease-free and overall survival in gastric cancer

		Disease-free suivival			Overall survival		
		
Characteristic	**No**.	HR	95%CI	p	HR	95%CI	p
Age(y)				.343			.583
< 60	95	1.00			1.00		
≥60	86	0.834	0.573-1.214		0.898	0.611-1.320	
Sex				.662			.950
Male	129	1.00			1.00		
Female	52	0.909	0.591-1.396		0.986	0.639-1.521	
Tumor size				.151			.232
≤4 cm	63	1.00			1.00		
> 4 cm	118	1.345	0.897-2.016		1.287	0.851-1.945	
pT stage				.001			.005
pT1	17	1.00			1.00		
pT2	35	1.549	0.558-4.301	.401	1.535	0.553-4.264	.411
pT3	119	3.546	1.437-8.750	.006	3.237	1.310-8.001	.011
pT4	10	4.738	1.547-14.506	.006	4.068	1.289-12.836	.017
pN stage				< .0001			.001
Negative	44	1.00			1.00		
Positive	137	2.617	1.538-4.451		2.389	1.401-4.075	
pTNM stage				< .0001			.001
I	26	1.00			1.00		
II	47	2.920	1.197-7.121	.018	2.860	1.173-6.973	.021
III	100	4.955	2.152-11.409	< .0001	4.455	1.930-10.281	< .0001
IV	8	7.369	2.470-21.981	< .0001	6.103	1.965-18.957	.002
p-mTOR				.008			.013
Negative	88	1.00			1.00		
Positive	93	1.667	1.146-2.454		1.644	1.112-2.430	

In multivariable analysis, lymph node metastasis (DFS, p = .018; OS, P = .038) and pT stage (DFS, p = .018; OS, P = .046) were significant independent prognostic factor for survival time. In addition, multivariable analysis (Table 3) also indicated a trend toward a correlation between p-mTOR expression and decreased survival, but the trend did not reach statistical significance (DFS, p = .059; OS, P = .070).

**Table 3:** Multivariable cox regression analysis of disease-free and overall survivals

		Disease-free suivival			Overall survival		
		
Factors	**No**.	HR	95%CI	p	HR	95%CI	p
p-mTOR				0.059			0.070
Negative	88	1.00			1.00		
Positive	93	1.452	0.986-2.138		1.443	0.970-2.147	
Infiltrating depth				0.018			0.046
pT1	17	1.00			1.00		
pT2	35	1.050	0.366-3.016	0.928	1.078	0.375-3.101	0.889
pT3	119	2.251	0.867-5.845	0.096	2.146	0.823-5.595	0.118
pT4	10	2.926	0.920-9.305	0.069	2.616	2.616-8.580	0.113
Lymph node status				0.018			0.038
Negative	44	1.00			1.00		
Positive	137	1.981	1.981-3.488		1.828	1.034-3.233	

### RAD001 inhibits proliferation and prevents p70S6K phosphorylation

**Figure 3:**
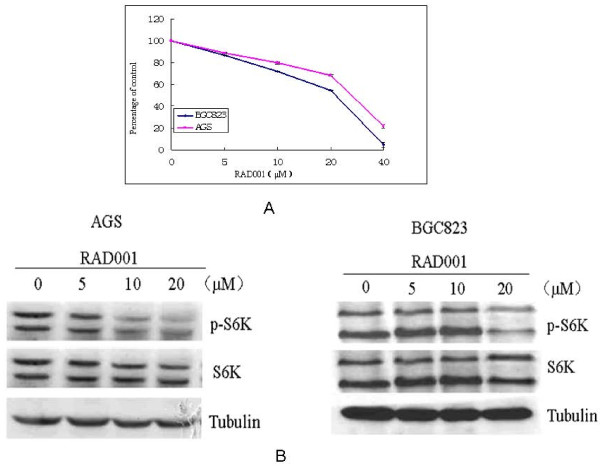
**(A) RAD001 inhibits proliferation of gastric cancer cells**. Gastric cancer cells were treated with the indicated concentrations of RAD001 in the presence of 10% FBS for 48 h. Cell viability was assessed by MTT assay. (B) Phosphorylation of p70s6K was significantly decreased in a dose-dependent manner in both AGS and BGC823 cells by the treatment with RAD001. Both cell lines were incubated with increasing dose of RAD001 for 48 hrs, cell lysates were subjected to western blot analysis with phosphorylated p-S6K and S6K antibodies.

To investigate the effects of RAD001 on the proliferation of human gastric cancer cell lines BGC823 and AGS, we performed proliferation assays with 48-hour exposure to RAD001. As a result, RAD001 generates significant inhibition on both cell lines in a dose-dependent manner. (Figure 3A).

Western blot experiments were performed with RAD001 at 0, 5, 10, 20 μM for 48 h. P70s6 kinase is an important downstream target of mTOR and its mTOR-dependent phosphorylation allows translation of ribosomal proteins. We examined the phosphorylation status of downstream target p70s6K by immunoblotting. As shown in Figure 3B, we found that treatment of both cell lines BGC823 and AGS with RAD001 significantly prevent phosphorylation of p70s6K in a dose-dependent manner.

### RAD001 induces G0/G1 cell cycle arrest and apoptosis in gastric cancer cells

**Figure 4:**
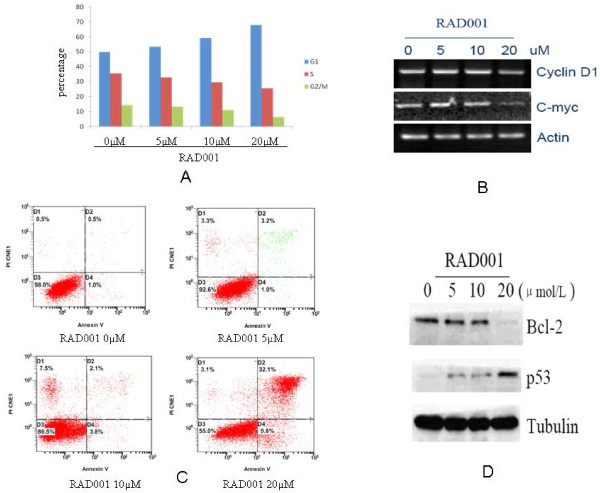
**RAD001 induces cell cycle arrest and apoptosis in BGC823 cells**. (A) BGC823 cells were exposed to various concentrations of RAD001 for 48 h. (B) RNA were extracted from cells. Cyclin D1 and C-myc expression level were detected by RT-PCR, actin as a loading control. (C) cells were incubated for 48 hrs with indicated doses of RAD001 before staining with annexin V-FITC. The percentages of apoptotic cells are displayed. (D) Cells were incubated for 48 hrs with indicated doses of RAD001. Cells were collected, lysed and subjected to Western blot analysis with Bcl-2 and p53 antibodies. GAPDH was used as a loading control.

To determine if the suppression in cell proliferation were due to G0/G1-phase arrest or apoptosis, cell-cycle distribution was analyzed by FCM. Figure 4 showed that treatment of BGC823 cells of with RAD001 for 48 hours resulted in a robust G1 arrest and promoted apoptosis. With the increasing dose of RAD001, more and more cells remained in G1-phase, while cell cycle progression into S phase was decreasing (Figure 4A). RT-PCR analysis revealed RAD001 dose-dependently decreased the mRNA level of Cyclin D1 and C-myc (Figure 4B).

Annexin-V assays showed that RAD001 significantly induced apoptosis in BGC823 cells. An increase in apoptosis rate was analyzed by FCM with the increase of the dose (Figure 4C). Western blot analysis showed RAD001 dose-dependently decreasing the protein level of Bcl-2 and increasing the protein level of p53 (Figure 4D). Similar results were observed in AGS cells (data not shown).

## Discussion

The mammalian target of rapamycin (mTOR) was discovered in the early 1990 s in studying the mechanism of action of rapamycin, which was originally found as an antifungal agent and was later recognized as anticancer properties [27]. As a Ser/Thr protein kinase, mTOR plays a key role in cellular growth and homeostasis [28]. The mTOR protein forms a complex with adaptor proteins, mTORC1 (mammalian target of rapamycin complex 1) and mTORC2. Activation of mTORC1 regulates cell growth by modulating protein synthesis, ribosome biogenesis and autophagy [29]. The mTORC2 pathway plays a key part of activating Akt, like PDK1(3-phosphoinositide-dependent protein kinase 1) and PI3K, a potential drug target for cancers in which there is Akt deregulation [26].

In the current study, we observed that 93 (51.4%) patients had positive expression of p-mTOR in 181 gastric cancer patients. Consistent with our result, in another study including 1072 patients, the expression of p-mTOR is 46.5% [30].

Previous studies have reported the prognostic significance of p-mTOR expression. Herberger et al [18]. identified p-mTOR to be an independent prognostic factor for death in patients with biliary tract adenocarcinoma. In their study, overall survival was significantly shorter in patients with p-mTOR-positive tumors as compared with patients with p-mTOR-negative tumors (P = .004). In gastric cancer, Yu et al. evaluated expression of p-mTOR in 1,072 gastric cancer patients using a tissue microarray, demonstrating overexpression of p-mTOR was an independent prognostic factor [30]. However, in a study included 109 patients with gastric adenocarcinomas who underwent a radical gastrectomy [31], Murayama et al. found neither cytoplasmic p-mTOR nor nuclear p-mTOR was independent prognostic factor, although they identified cytoplasmic p-mTOR expression was associated with poorer survival and correlated with the depth of tumour invasion, lymph nodes and tumour stage. In the present study, we confirmed that pT classification and lymph node status are independent prognostic indicator of clinical outcome in gastric cancer patients by multivariable analyses. However, because most patients present with locally advanced disease, other secondary prognostic markers, particularly p-mTOR, may be of use [13]. Our results clearly show that p-mTOR expression is correlated strongly with poor overall and disease-free survival in univariable analyses with a trend toward correlation in multivariable analyses.

Consistent with our current study, several studies have also recognized the antiproliferative effects of mTOR inhibitors in gastric cancer cells. For example, Lang et al [21]. identified rapamycin could inhibit gastric cancer cell growth in both a subcutaneous tumor model and in an experimental model. Their results highlight the suitability of mTOR inhibitors to be used in an antiangiogenic context for therapy of gastric cancer.

However, RAD001 is a new mTOR inhibitor as anticancer agent. The mechanism about RAD001 in gastric cancer is not clear. Cejka et al. showed the antiangiogenic activity of RAD001 combined with a high dose of cyclophosphamide revealed synergistic antitumor activity against gastric cancer [32]. They have also found that RAD001 decreased proliferation and attenuated production of HIF-1α as well as VEGF in gastric cancer cells in vitro [33]. In the current study, our data showed that RAD001-therapy attenuated phosphorylation of p70S6K and markedly inhibited the proliferation of gastric cancer cells through cell-cycle arrest and apoptosis in vitro. We found RAD001 induced the G0/G1 phase arrest, which was associated with decreased cyclin D1 and C-myc. Moreover, we showed for the first time that RAD001 as a new mTOR inhibitor dose-dependently induced apoptosis in gastric cancer cells by Annexin V assays. This effect may be dictated by the cellular context and downstream targets including P53 and Bcl-2. These findings might further explain the efficacy of RAD001.

## Conclusion

In summary, we conducted systematical research to mTOR in gastric cancer, indicating the relationships the expression with the prognosis value of activated mTOR (p-mTOR) in vivo, the effect of RAD001 and the part of mechanisms in gastric cancer cells in vitro. Our findings indicated that p-mTOR would serve as a potential biological marker to identify a subgroup of gastric cancer patients of poor prognosis. It also showed that RAD001 was a promising agent for the treatment of gastric cancer with inducing cell cycle arrest and apoptosis by down-regulating expression of C-myc, CyclinD1 and Bcl-2, up-regulating expression of P53. Further studies are required to elucidate the role of the activation of mTOR and eventually to propose mTOR as a concrete target for gastric cancer therapy.

## Competing interests

The authors declare that they have no competing interests.

## Authors' contributions

DZX and QRG designed the research, final data analysis and drafted the manuscript. YT carried out the molecular studies. XJF and MYC assessed all slides independently. ZWZ, WL, YBC, XWS, YXG and YFL collected the gastric cancer tissues. TYL and YQZ supervised all the work. All authors read and approved the final version of the manuscript.

## Note

Grant Support: Supported by National High Technology Research and Development (863) Program of China

## Pre-publication history

The pre-publication history for this paper can be accessed here:

http://www.biomedcentral.com/1471-2407/10/536/prepub
